# Effectiveness of school-based sexual and reproductive health education among adolescent girls in Urban areas of Odisha, India: a cluster randomized trial

**DOI:** 10.1186/s12978-023-01643-7

**Published:** 2023-07-19

**Authors:** G. Alekhya, Swayam Pragyan Parida, Prajna Paramita Giri, Jasmina Begum, Suravi Patra, Dinesh Prasad Sahu

**Affiliations:** 1 Department of Community Medicine and Family Medicine, AIIMS Bhubaneswar, 3rd Floor, Academic Block, Bhubaneswar, Odisha 751019 India; 2 Department of Obstetrics and Gynaecology, AIIMS Bhubaneswar, Bhubaneswar, Odisha India; 3 Department of Psychiatry, AIIMS Bhubaneswar, Bhubaneswar, Odisha India; 4 WHO NTEP, Bhubaneswar, Odisha India

**Keywords:** Adolescence, Adolescent girls, Sexual and reproductive health, Adolescent sexual and reproductive health, School health, Health education, Randomized trials

## Abstract

**Background:**

Various studies revealed that adolescent girls have limited knowledge pertaining to sexual and reproductive health (SRH). The current study assessed the effectiveness of SRH education among adolescent girls in urban areas of Odisha, India.

**Methods:**

The study design was a cluster randomized trial, where the clusters composed of eight Odia (regional language) medium government girls’ high schools in Bhubaneswar, the capital city of the state of Odisha, India**.** For the selection of study participants, adolescent girls who were studying in the ninth and tenth standards were recruited from each school. Eight schools were randomized through restrictive randomization at a 1:1 ratio, with four schools each in the intervention and control arm. Baseline and end-line assessments were done using a pre-tested, semi-structured questionnaire. Following baseline assessment, an intervention was given with the help of handbooks developed by the study authors to the schools in the intervention arm. Outcomes included change in knowledge, attitude and practices pertaining to SRH.

**Results:**

In our study at baseline, there were a total of 790 students, where 469 (59.4%) students were in the intervention arm, and 321 (40.6%) students were in the control arm. At baseline, only 282 (60.1%) in the intervention arm and 171 (53.3%) in the control arm were aware that physical bodily changes due to puberty were normal. After the intervention, there was a statistically significant increase in knowledge in intervention group 367 (94.8%) (p-value < 0.001). Most students used sanitary pads as absorbent, 97.2% in the intervention group and 98.4% in the control group. However, after the intervention, the use of other absorbents reduced to zero in the intervention group with a statistically significant difference (p < 0.05). The number of students having awareness on different methods of contraception increased from 51 (10.9%) to 337 (87.1%) in the intervention arm (p < 0.001), and of those having awareness on STIs/RTIs increased from 177 (38.2%) to 371 (96.1%) in the intervention group (p < 0.001).

**Conclusion:**

From our study, there is a significant proportional change in knowledge, attitude, and practices pertaining to SRH. Our study recommends policymakers and program managers for the implementation of comprehensive SRH in the regular school curriculum.

*Trial registration* CTRI/2021/01/030490, registered on January 15, 2021. Prospectively registered at https://ctri.nic.in/Clinicaltrials/login.php

## Plain language summary

Adolescent girls lack adequate knowledge pertaining to sexual and reproductive health, for which they face various issues such as teenage pregnancy, unsafe abortions and sexually transmitted infections. Hence the authors conducted this study to assess the effectiveness of school-based sexual and reproductive health education among adolescent girls in Odisha state of India in terms of improving knowledge, attitude and practices pertaining to reproductive health. A total of eight vernacular (Odia language) medium schools were selected for the conduct of the study, and the schools were randomly assigned where four schools received SRH education, and the other four received no intervention. A baseline assessment pertaining to SRH was done among adolescent girls studying in ninth and tenth classes of all eight schools, and then education was given to the four schools in the intervention arm. Students were educated on topics such as puberty, menstrual health, pregnancy, contraception and STIs/RTIs with the help of handbooks prepared by the authors. After 3 months of providing education, an endline assessment was done for the adolescent girls in all eight schools. There was an increase in knowledge, attitude and practices pertaining to SRH among the students who received education when compared to those who didn’t. Hence our study recommends policymakers and program managers include SRH education in the regular school curriculum.

## Background

As per United Nations Children’s Fund (UNICEF), there are approximately 1.3 billion adolescents worldwide, with 90% of them living in developing countries. Among these, there are around 880 million adolescent girls [1]. Gender inequality has persisted in the communities marginalizing adolescent girls who are victims of pernicious social norms affecting their ability to make decisions regarding education, work, marriage and social relationships [2]. The United Nations Population Fund (UNFPA) defines Sexual and Reproductive health (SRH) as a state of complete physical, mental, and social well-being related to the reproductive system [3]. Literature shows that adolescent girls lack adequate knowledge on SRH, for which they face issues such as early pregnancy and childbirth, abortion, violence, unintended pregnancies, maternal mortality, reproductive tract infections (RTIs) and sexually transmitted diseases (STDs) [4]. Around twelve million girls aged 15–19 years and seven million girls under 15 years give birth each year in developing countries. Complications arising due to pregnancy and childbirth are the leading cause of death for girls aged 15–19 years globally [5]. Each year around 39,000 child marriages happen every day [6]. Available data suggest that adolescent mothers succumb to depression compared to non-pregnant peers and adult mothers [7]. The Sustainable Development Goal (SDG) 3.7 states that by the year 2030, there should be universal access to sexual and reproductive health care services, including family planning, education, and integration of reproductive health into national programs. Also, SDG 5 focuses on gender equality by empowering women and girls, but data suggests only 57% of women aged 15–49 years make informed decisions regarding sexual and reproductive health care [8]. International organizations such as the World health organization (WHO), United Nations Children’s Fund (UNICEF), and the Lancet Commission highlight the need to prioritize adolescents in achieving the SDGs [9].

India, with a population of 253 million, has the world’s largest adolescent population [10]. In 2014, the Government of India (GOI) launched the “Rashtriya Kishor Swasthya Karyakram (RKSK)” to provide services to adolescents, including SRH services [11]. However, studies have shown that service utilization remains poor, and adolescents are often unaware of these services [12]. In Odisha state, under the RKSK program, Adolescent friendly health clinics (AFHCs) are operational at Urban Primary Health Centers (PHC), with adolescent health counsellors providing clinical, counselling and outreach services to schools, colleges and youth clubs [13]. However, in a scoping review measuring adolescent-friendly health services in India, in Odisha state, community health workers had more knowledge about adolescent health programs than the teachers and the prevalence of SRH knowledge was low in the adolescent community [14]. As per the latest National family health survey-5 (NFHS-5), data shows that 6.8% of women aged 15–19 have begun childbearing, and 23.3% of women aged 20–24 married before the age of 18. In Odisha, a state in India, the prevalence of teenage pregnancy is 7.6%, and 20.5% of married women were below 18 years of age, similar to the national data [15]. These rates are far from the recommendations of the Lancet Commission on adolescent health, which states the prevalence of teenage pregnancy to be less than 1 per cent by the year 2030 [16]. SRH education in India has not been given much importance due to existing taboos. A report on sexuality education in India by Youth Coalition for Sexual and reproductive rights stated that higher secondary schools do not have SRH education in the curriculum; however, private schools had the liberty to choose for inclusion of SRH education in the curriculum, but no attempts have been made from public schools mainly vernacular government schools [17]. This report was made in accordance with the International Conference on Population and Development (ICPD), which states governments are obliged to provide comprehensive sexuality education for youth to make informed decisions [17]. But some Indian states, such as Maharashtra, Gujarat, Karnataka, Rajasthan, Kerala, Goa, Madhya Pradesh, and Chhattisgarh, have banned sex education [18]. Studies conducted across various states in India assessed the knowledge, attitude and practices (KAP) related to SRH among adolescent girls and showed poor awareness among adolescents [19]. While some interventional studies assessed SRH education among adolescent girls in India, most of them were non-randomized studies [20, 21]. Only one randomized study compared conventional education delivered by nurses with peer education among school-going adolescent girls in Punjab state, which found that both approaches improved knowledge [22]. However, no studies assessed the effectiveness of a comprehensive SRH intervention package with a control arm.

Schools act as a platform for providing educational interventions, given the concentration of the adolescent population at schools and the ease of access to health promotion in poor communities without effective health systems [23]. The authors of the study aimed to assess the effectiveness of school-based comprehensive SRH education in improving knowledge, attitude, and practices related to puberty, menstrual health, pregnancy, contraception, and RTIs/STDs among adolescent girls studying in vernacular (Odia medium) secondary girls’ high schools in Odisha, India.

## Methods

### Study setting

The current study was conducted in Bhubaneswar, the capital city of Odisha state in India. Schools in India are broadly categorized into four types based on the enrollment of students: Lower primary school (classes 1 to 5), Upper primary school (classes 6th and 7th), High school (classes 8th, 9th and 10th), and Higher secondary school (class 11th and 12th). Each state in the country runs its own Department of Education. Schools in each state are of three kinds: government schools, privately owned schools, and schools that are provided grant-in-aid by the government [24]. After reviewing the syllabus, it was found that there was not much emphasis made on SRH in the state curriculum. Hence schools under the Odisha State Board of Education were selected to conduct the present study. The study was conducted among vernacular (Odia medium) girls’ high schools in Bhubaneswar city. For the conduct of the study, permission was obtained from District Education Officer (DEO), Khordha. There are a total of eight vernacular girls’ high schools in Bhubaneswar City, and all the schools were included in the study.

### Study design and sampling strategy

The study design was a cluster randomized trial conducted from May 2020 to April 2022. During this period, there was an ongoing COVID-19 pandemic. The schools remained closed as per guidelines from the Government of Odisha (GOO). The schools conducted online classes using various virtual platforms such as Zoom meetings, Google Meet, and YouTube. Permission was taken to assess the students through online and offline modes when required and to give education to school students through both online and offline modes depending upon the school closure. Written informed consent was taken from parents of adolescent girls, and assent was sought from adolescent girls.

For sample size calculation, as per a study conducted in Gujarat in the year 2017, the proportion of participants having awareness about STIs varied between 23 and 29% [25]. Considering a total average of 26% as a baseline and assuming a 15% increase at end-line assessment, the sample size was calculated using nMaster software. The total sample size obtained was 345, with α at a significance level of 0.05 and the power of study being 91, and with design effect 2. Considering attrition of ten per cent, the calculated sample size was 380 per arm.

The sampling frame included all eight government girls’ high schools (GHS) in Bhubaneswar, Odisha. Schools were considered as clusters, and restrictive randomization was done to randomize eight schools at a ratio of 1:1, with four schools in each intervention and control arm. For the selection of study participants as per protocol, systematic random sampling was to be done among adolescent girls studying in ninth and tenth classes. However, due to the COVID-19 pandemic, all those who responded to the baseline questionnaire were included, and an amendment in protocol was done regarding the same and was approved by Institute Ethics Committee (IEC), AIIMS Bhubaneswar. Outcome variables included changes in KAP pertaining to domains such as puberty, menstrual health, pregnancy and contraception, STIs/RTIs, and HIV/AIDS.

### Baseline assessment

A pre-tested semi-structured questionnaire was developed, which was adopted from the Illustrative questionnaire for interview surveys with young people, World Health Organization [26]. The questionnaire consists of components such as socio-demographic details of the students and KAP in domains such as puberty, menstrual health, pregnancy, contraception, STIs/RTIs, and HIV/AIDS. The questions on KAP related to various domains of SRH were prepared with reference to the International technical guidance on sexuality education by the UNESCO education sector [27]. The document includes a section on key concepts pertaining to different topics, including SRH, where learners should acquire knowledge, attitudes and skills based on these key concepts. The knowledge component focused on evaluating adolescent girls’ understanding of pubertal changes, including the concept of the menstrual cycle, the process of pregnancy, risks associated with teenage pregnancy, the concept of contraception and its various methods, as well as awareness of RTI/STIs, and HIV/AIDS. The attitude component aimed to gauge adolescent girls’ beliefs regarding pubertal changes, menstruation hygiene management, their opinions on sex education, and their perception of how an HIV-infected person should be treated. The practice component primarily examined menstrual hygiene practices. Due to the cultural context of the Indian setting, the authors made a decision not to include questions on contraceptive practices in the assessment. The questionnaire was developed in English and later translated into the local language (Odia) with the help of a professional translator. Also, a back translation of the questionnaire from Odia to English was done to test the accuracy of the translation. The questionnaire was pre-tested among 36 students who were not a part of the selected schools. The questionnaire for the baseline assessment of students was delivered to the students through online Google forms. The assessment was done in August–September 2021 during the second wave of the COVID-19 pandemic. Google forms were delivered in WhatsApp groups of seven selected schools after obtaining consent from the school principal and teachers at respective schools. Of eight schools, one was a residential school, where students who resided in rural areas lacked access to smartphones. In the month of August 2021, schools were re-opened, and assessment of the students at residential school was done through offline forms.

### Intervention

A literature search was done for the preparation of an intervention package on SRH education. The intervention package consisted of handbooks covering the following topics.

1. Handbook Part 1—Adolescent health statistics, Female reproductive system, Puberty, and Menstrual Health
2. Handbook Part 2—Pregnancy, Contraception, STIs/RTIs, and HIV/AIDS

The principal investigator has done a literature search through various websites of WHO and UNICEF, which focused on adolescent health. The relevant documents were then downloaded. The content required as per need has been retrieved from the documents and has been included in handbooks. Also, various Indian documents and modules pertaining to adolescent health, textbooks of school, and Obstetrics and Gynaecology, were referred for handbook preparation. The handbooks were developed in the English language. Since the vernacular language is Odia, the handbooks were translated into Odia by a professional Odia translator. The translated handbooks were given to two medico-social workers (MSWs) separately to verify comprehension of the language. The intervention was given in the months of November and December 2021 for the schools in the intervention arm. Owing to the COVID-19 pandemic, schools were running only half—a day, and accordingly, intervention timing was fixed. The author (AG) visited all the four schools in the intervention arm to provide SRH education to school-going adolescent girls. In each school, the intervention included three sessions. Three sessions were done on 3 consecutive days in each school. Each session lasted for about 2 h. The intervention was given to adolescent girls studying in ninth and tenth classes separately. In the first session, education was given on topics such as the female reproductive system, puberty, and menstrual health with the help of the first part of the handbook. The second session included the topics of part 2 of the handbook, i.e., pregnancy, contraception, STIs/RTIs, and HIV/AIDS. The intervention included a PowerPoint presentation with the help of a projector, brainstorming sessions, and a discussion of case scenarios. The third session was an interactive session between the students and teachers, and various doubts were cleared.

**Fig. 1:**
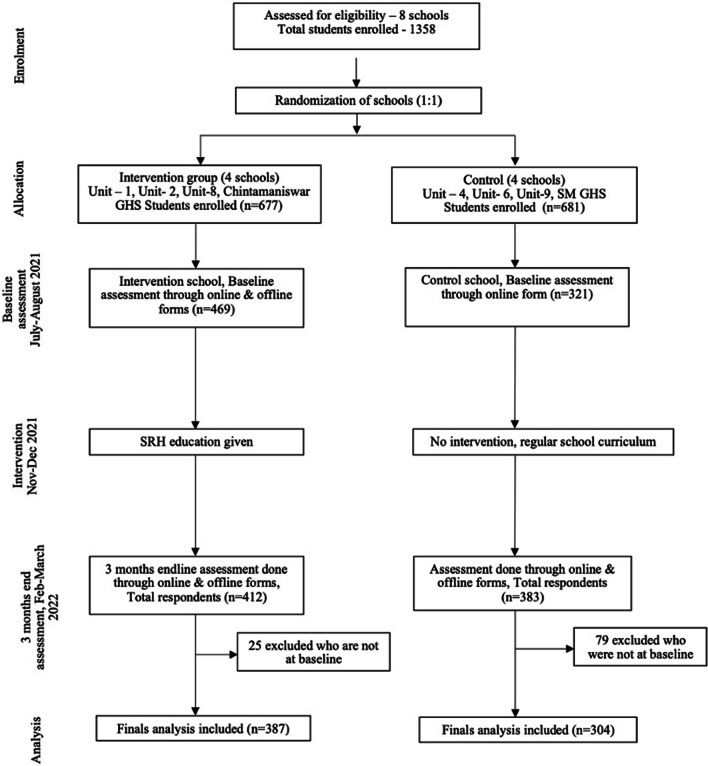
CONSORT flow diagram

Following the intervention, an endline assessment was conducted 3 months later among all eight schools using online Google forms. The assessment of each school was done on 8 different days between the months of February and March 2022. Once the endline assessment was completed, the same intervention was provided to students in the control arm. The flow of the study design is depicted in Fig. 1.

### Data entry and statistical analysis

The recorded responses were exported to Microsoft (MS) Excel except in one school where data was collected through offline forms and entered manually into MS Excel. The data entry was done simultaneously on the same day of the collection of data by the principal investigator. Statistical analysis was done using International Business Machines (IBM)—Statistical Package for social sciences (SPSS) version 26. Descriptive data on socio-demographic details of students are presented in percentage or proportion. Quantitative variables such as the age of the students are expressed as mean and standard deviation. To assess the effectiveness of the intervention, the proportion of students in KAP of all domains was considered. The proportional change was measured among the intervention and control groups before and after the intervention by using the Chi-square test. With the cells having a count less than five, Fischer exact test was used. A p-value of < 0.05 was considered significant.

## Results

The baseline sample included a total of 790 students from eight government vernacular (Odia medium) girls’ high schools. Among 790 students, 469 (59%) were in the intervention arm, and 321 (41%) were in the control arm. The mean age of students in the intervention arm was 14.4 ± 0.92 years, whereas in the control arm was 14.4 years ± 0. 8 years. The majority of students’ parents, both mother and father, studied till high school (37% and 34.3% respectively), and occupations included mothers being homemakers (91%) and fathers either clerk/shopkeeper/worker (39.7%). There was no statistically significant difference in baseline characteristics between intervention and control groups (Table 1).

**Table 1:** Baseline socio-demographic characteristics of intervention and control group (N = 760)

Variables	Categories	Total N = 790, n (%)	Intervention N = 469, n (%)	Control N = 321 (%)	p-value
Class	9th	414 (52.4)	255 (54.4)	159 (49.5)	0.181
	10th	376 (47.6)	214 (45.6)	162 (50.5)	
Age group	≤14 years	441 (55.8)	257 (54.8)	184 (57.3)	0.483
	> 14 years	349 (44.2)	212 (45.2)	137 (42.7)	
Mother education	Illiterate	61 (7.7)	39 (8.3)	22 (6.9)	0.625
	Class (1–5)	125 (15.8)	79 (16.8)	46 (14.3)	
	Class (6–8)	84 (10.6)	46 (9.8)	38 (11.8)	
	Class (9–10)	292 (37.0)	173 (36.9)	119 (37.1)	
	Class (11–12)	166 (21.1)	94 (20.1)	72 (22.4)	
	Bachelors	43 (5.4)	24 (5.1)	19 (5.9)	
	Masters	19 (2.4)	14 (3.0)	5 (1.6)	
Father education	Illiterate	40 (5.1)	26 (5.6)	14 (4.4)	0.069
	Class (1–5)	84 (10.6)	42 (9.0)	42 (13.1)	
	Class (6–8)	82 (10.4)	46 (9.8)	36 (11.2)	
	Class (9–10)	271 (34.3)	170 (36.3)	101 (31.5)	
	Class (11–12)	197 (25.0)	124 (26.5)	73 (22.7)	
	Bachelors	79 (10.0)	37 (7.9)	42 (13.1)	
	Masters	36 (4.6)	23 (4.9)	13 (4.0)	
Occupation of mother	Homemaker	719 (91.0)	425 (90.6)	294 (91.6)	0.576
	Unskilled worker	16 (2.0)	10 (2.1)	6 (1.9)	
	Skilled worker	31(3.9)	22 (4.7)	9 (2.8)	
	Clerical/shop/worker	12 (1.5)	6 (1.3)	6 (1.9)	
	Semi-professional	11 (1.4)	6 (1.3)	5 (1.6)	
	Professional	1 (0.2)	0 (0.0)	1 (0.2)	
Occupation of father	Unemployed	16 (2.0)	2 (0.4)	14 (4.3)	0.095
	Unskilled worker	155 19.6)	96(20.4)	59 (18.4)	
	Semi-skilled worker	13 (1.6)	6 (1.2)	7 (2.1)	
	Skilled worker	154 (19.7)	90 (19.1)	64 (20.0)	
	Clerical/shop/worker	311 (39.3)	192 (41.3)	119 (37.1)	
	Semi-professional	121 (15.3)	74 (15.7)	47 (14.6)	
	Professional	20 (2.5)	9 (1.9)	11 (3.5)	

Table 2 shows KAP pertaining to puberty and menstrual health among intervention and control arms at baseline and 3 months endline. At baseline, only 282 (60.1%) in the intervention arm and 171 (53.3%) in the control arm were aware that physical bodily changes due to puberty were normal. After the intervention, there was a statistically significant increase in knowledge in the intervention arm to 94.8% (n = 367) (p < 0.01). At baseline, 297 (63.3%) in the intervention arm and 215 (67%) in the control arm considered menstruation as a good thing, and after the intervention, there was a statistically significant (p < 0.01) increase in knowledge in the intervention group, where 362 (93.5%) considered menstruation a good thing in comparison to the control arm 219 (72%). Most students used sanitary pads as absorbent, 97.2% in the intervention group and 98.4% in the control group. However, after the intervention, the use of other absorbents was reduced to zero in the intervention group (p < 0.05).

**Table 2:** Knowledge and attitude pertaining to puberty and menstrual health in intervention and control group at baseline and 3 months endline

Characteristics	Response	Baseline			Three months endline		
		Intervention	Control	P value	Intervention	Control	P value
		N = 469, n (%)	N = 321, n (%)		N = 387, n (%)	N = 304, n (%)	
Puberty							
Physical bodily changes occurring due to puberty: do you take it as normal	Yes	282 (60.1)	171 (53.3)	0.089	367 (94.8)	188 (62.0)	< 0.01
	No	85 (18.1)	77 (24.0)		12 (3.1)	60 (19.8)	
	Don't know	102 (21.7)	73 (22.7)		8 (2.1)	55 (18.2)	
Do pubertal changes vary between individuals	Yes	154 (32.8)	106 (33.0)	0.016	342 (88.4)	145 (47.9)	< 0.01
	No	44 (9.4)	51 (15.9)		18 (4.7)	44 (14.5)	
	Don't know	271 (57.8)	164 (51.1)		27 (7.0)	114 (37.6)	
Do you feel comfortable when puberty-related topics are discussed in class?	Yes	282 (60.1)	198 (61.7)	0.66	352 (91.0)	155 (51.0)	< 0.01
	No	187 (39.9)	123 (38.3)		352 (91.0)	155 (51.0)	
Menstrual health							
Is menstruation a good thing	True	297 (63.3)	215 (67.0)	0.293	362 (93.5)	219 (72.0)	< 0.01
	False	78 (9.9)	34 (10.6)		14 (3.6)	25 (8.2)	
	Can't say	200 (25.3)	72 (22.4)		11 (2.8)	60 (19.7)	
Females who have attained menarche/have menses can become pregnant	True	280 (59.7)	208 (64.8)	0.148	351 (90.7)	252 (82.9)	< 0.01
	False	27 (5.8)	12 (3.7)		11 (2.8)	7 (2.3)	
	Can't say	162 (34.5)	101 (31.5)		25 (6.5)	45 (14.8)	
Excessive bleeding during menstruation leads to anemia	True	179 (38.2)	142 (44.2)	0.158	335 (86.8)	219 (72.0)	< 0.01
	False	89 (19.0)	62 (19.3)		31 (8.0)	26 (8.6)	
	Can't say	201 (42.9)	117 (36.4)		20 (5.2)	59 (19.4)	
Activities during menstruation need to be restricted	True	239 (51.1)	194 (60.4)	0.032	87 (22.5)	100 (32.9)	< 0.01
	False	85 (18.2)	50 (15.6)		286 (74.1)	55 (18.1)	
	Can't say	144 (30.8)	77 (24.0)		13 (3.4)	149 (49.0)	
Diet during menstruation needs to be restricted	True	218 (46.6)	153 (47.7)	0.166	80 (20.7)	141 (46.4)	< 0.01
	False	121 (25.9)	97 (30.2)		297 (76.7)	98 (32.2)	
	Can't say	129 (27.6)	71 (21.1)		10 (2.6)	137 (45.1)	
Do you attend school during periods?	Yes	402 (85.9)	259 (80.7)	0.051	366 (94.6)	271 (89.1)	0.008
	No	66 (14.1)	62 (19.3)		21 (5.4)	33 (10.9)	
Girls should be taught about menstrual hygiene in schools	Yes	452 (96.6)	313 (97.5)	0.457	384 (99.25)	296 (97.4)	0.067
	No	16 (3.4)	8 (2.5)		3 (0.8)	8 (2.6)	
Which absorbent do you use during menstruation?	Sanitary Pad	456 (97.2)	316 (98.4)	0.741	385 (99.0)	295 (97.0)	< 0.01
	Cloth	2 (0.4)	1 (0.3)		2 (0.5)	2 (0.7)	
	Others	11 (0.9)	4 (0.6)		0 (0.0)	2 (0.7)	
How many times do you change your pad or cloth?	Once a day	30 (6.4)	18 (5.6)	0.041	8 (2.1)	15 (4.9)	< 0.01
	2–3 times a day	241 (51.4)	194 (60.4)		197 (50.9)	186 (61.2)	
	More than three times a day	198 (42.2)	109 (34.0)		182 (47.0)	103 (33.9)	
	No	172 (36.75)	54 (16.8)		16 (4.1)	25 (8.2)	
Do you wash your hands with soap after changing your sanitary napkin?	Yes	458 (97.7)	316 (98.4)	0.44	382 (98.7)	296 (97.4)	0.198
	No	11 (2.3)	5 (1.6)		5 (1.3)	8 (2.6)	

Table 3 shows KAP pertaining to pregnancy, contraception, RTIs/STIs and HIV/AIDS among intervention and control arms at baseline and 3 months endline. Only 78 (16.6%) in the intervention group and 67 (20.9%) in the control group were aware that pregnancy could occur with a single sexual act; however, after the intervention, it increased to 81.3% in the intervention arm and in the control arm it was 16.8% (p < 0.01). Awareness on contraception was only 134 (28.6%) in the intervention arm and 113 (35.2%) in the control arm. After the intervention, there was a statistically significant increase, where 360 (93%) in the intervention arm and 105 (34.5%) in control became aware that pregnancy could be prevented by using contraceptives. Awareness on different methods of contraception was 51 (10.9%) in the intervention arm and 39 (12.1%) in the control. After the intervention, the awareness increased, where 337 (87.1%) in the intervention arm became aware of different methods of contraception (p < 0.01). At baseline, 177 (38.2%) in the intervention arm and 116 (36.4%) in the control arm were aware of STIs/RTIs; after the intervention, awareness increased to 96.1% in the intervention arm, and 44.1% in the control group were aware (p < 0.01). Regarding awareness on HIV/AIDS at baseline, students in the control arm (50.2%) were more aware when compared to the intervention arm (42.6%) with statistical significance (p < 0.05). However, after the intervention, awareness increased to 95.6% in the intervention arm and in the control arm, only 44.1% were aware (p < 0.01).

**Table 3:** Knowledge and attitude pertaining to pregnancy, contraception, RTIs/STIs and HIV/AIDS among intervention and control arms at baseline and 3 months endline

Characteristics	Response	Baseline			Three months endline		
		Intervention	Control	P value	Intervention	Control	P value
		N = 469, n (%)	N = 321, n (%)		N = 387, n (%)	N = 304, n (%)	
Pregnancy and contraception							
Pregnancy can occur with one sexual act	True	78 (16.6)	67 (20.9)	0.312	314 (81.3)	51 (16.8)	< 0.01
	False	53 (11.3)	33 (10.3)		22 (5.7)	24 (7.9)	
	Can't say	338 (72.1)	221 (68.8)		50 (13.0)	229 (75.3)	
Early pregnancy before 18 years increases the chance of maternal death	True	181 (38.6)	145 (45.2)	0.182	354 (91.5)	120 (39.5)	< 0.01
	False	25 (5.3)	15 (4.7)		10 (2.6)	17 (5.6)	
	Can't say	263 (56.1)	161 (50.2)		23 (5.9)	167 (54.9)	
Pregnancy can be prevented by using contraceptives	True	134 (28.6)	113 (35.2)	0.131	360 (93.0)	105 (34.5)	< 0.01
	False	24 (5.1)	13 (4.0)		6 (1.65)	8 (2.6)	
	Can't say	311 (66.3)	195 (60.7)		21 (5.4)	191 (62.8)	
Are you aware of different methods of contraception?	Yes	51 (10.9)	39 (12.1)	0.579	337 (87.1)	22 (7.2)	< 0.01
	No	418 (89.1)	282 (87.9)		50 (12.9)	282 (92.8)	
Are you aware of the emergency contraceptive pill?	Yes	62 (13.2)	42 (13.1)	0.956	348 (90.2)	48 (15.6)	< 0.01
	No	407 (86.8)	279 (86.9)		38 (9.85)	256 (84.2)	
Girls should be taught sex education	True	334 (71.2)	256 (79.8)	0.023	367 (95.1)	217 (71.4)	< 0.01
	False	10 (2.1)	6 (1.9)		2 (0.5)	9 (3.0)	
	Can't say	125 (26.7)	59 (18.4)		17 (4.4)	78 (25.7)	
STIs/RTIs and HIV/AIDS							
Have you heard about STIs/RTIs?	Yes	177 (38.2)	116 (36.4)	0.596	371 (96.1)	134 (44.1)	< 0.01
	No	286 (61.8)	203 (63.6)		15 (3.9)	170 (55.95)	
Are you aware of HIV/AIDS?	Yes	200 (42.6)	161 (50.2)	0.037	369 (95.6)	143 (47.0)	< 0.01
	No	269 (57.4)	160 (49.8)		17 (4.4)	161 (53.0)	
HIV is transmitted through sexual contact	True	124 (26.4)	112 (34.9)	0.039	343 (88.6)	99 (32.6)	< 0.01
	False	19 (4.1)	12 (3.7)		16 (4.1)	16 (5.3)	
	Can't say	326 (69.5)	197 (61.4)		28 (7.2)	189 (62.2)	
Condoms protect against STI/HIV	True	96 (20.5)	70 (21.8)	0.662	354 (91.5)	67 (22.0)	< 0.01
	False	17 (3.6)	15 (4.7)		4 (1.0)	7 (2.3)	
	Can't say	356 (75.9)	236 (73.5)		29 (7.5)	230 (75.7)	
A person infected with HIV should be removed from school	True	80 (17.1)	68(21.2)	0.02	44 (14.5)	33 (8.5)	< 0.01
	False	71(15.1)	74(23.1)		67 (22.0)	326 (84.2)	
	Can't say	318(67.8)	179(55.8)		193 (63.5)	28 (7.2)	

## Discussion

The current study assessed the effectiveness of SRH education among vernacular school-going adolescent girls. At baseline assessment, adolescent school-going girls lacked adequate knowledge pertaining to SRH. However, post-intervention, there was a significant increase in KAP in the intervention arm when compared to the control arm. There are studies conducted across various countries to assess the effectiveness of SRH education among adolescent girls, with outcomes having an increase in knowledge pertaining to SRH [28–34]. In India, only one randomized trial was conducted assessing peer education and conventional educational strategies for improving SRH. However, both were effective, and peer education was found to be more cost-effective [22]. To our knowledge, this is the first cluster randomized trial in India to assess the effectiveness of SRH education among school-going adolescent girls.

In our study, the intervention was provided by a community physician, with the intervention delivered using PowerPoint presentations and handbooks. The KAP increased in all domains, such as puberty, menstrual health, pregnancy, contraception, RTIs/STIs and HIV/AIDS following the intervention. In our study at baseline, about 50–60% of adolescent girls were aware of pubertal changes, and forty per cent of students said they were not comfortable when puberty-related topics were discussed. After the intervention, awareness on puberty increased to 94.8%. Our finding was similar to an interventional study conducted in Kerala among school-going adolescent girls, where pubertal awareness increased from 32 to 83.9%, where intervention was delivered through interactive and quiz sessions [20]. Hence, there is a need for sensitizing students regarding pubertal changes, and training of schoolteachers would be helpful. In our study, 60% of adolescents felt restriction of activities during menstruation. However, it was reduced to 22% following the intervention, indicating that there are still myths about menstruation. The majority of students attended schools during menstruation; Our study finding could be due to the provision of sanitary napkins free of cost under the “Khushi scheme” by the government of Odisha [35]. It can be inferred that the provision of sanitary napkins facilitated the attendance of adolescent girls, which can be correlated with a study conducted in Gujarat, where the provision of sanitary napkins reduced absenteeism from 24 to 14% [21]. In our study, most of the students, both in the intervention and control arm, felt that menstrual hygiene should be taught in schools as vernacular government girls’ high schools did not provide education on menstrual hygiene management (MHM) when imparted priorly at an earlier age before attaining menarche will inculcate healthy menstrual hygiene practices among adolescent girls.

In our study at baseline, only 20–30% of adolescent girls were aware of pregnancy and contraception. However, after the intervention, knowledge on pregnancy, different contraception and emergency contraception methods increased to 90%. In an interventional study conducted in Gujarat, knowledge pertaining to contraception increased from 10 to 32% among adolescent boys and girls [25]. In the study done in Kerala, adolescent girls who were unaware of the prevention of pregnancy reduced from 63 to 13% [20]. The study done in Kerala was about a decade ago, but still, no efforts have been made to provide a comprehensive SRH curriculum. Our study noted that at baseline, students were more aware of HIV/AIDS as a disease but were not aware of RTIs/STIs. This finding may be due to HIV/AIDS being a more deadly disease than RTIs/STIs and awareness being provided on various platforms such as social media and celebrating World AIDS Day. Also, at baseline, awareness of HIV/AIDS was more in the control arm when compared to intervention, and this finding was statistically significant. This could be because one of the intervention schools was a residential school with students hailing from rural areas, whereas students from control schools were residing in urban areas. However, after the intervention, awareness of RTIs/STIs increased to 90%. Our study finding was similar to a randomized trial conducted in countries such as Zimbabwe among secondary school students, where awareness of RTIs/STDs increased from 20 to 96% [36] and in the study conducted in Gujarat state, awareness regarding STIs increased from 29 to 32% [25]. Hence, by imparting education on pregnancy and contraception, adolescent girls can make informed choices regarding their sexual health. Moreover, in the study, about two-thirds of students favoured sex education to be taught in school. As mentioned earlier, in India, various states have banned sex education, but evidence from various studies conducted in India showed parents, teachers, and students favoured sex education in school and to be provided by doctors [37–39]. Also, evidence reviews from various countries by UNESCO have stated that CSE should be a holistic strategy where young people shape their sexual and reproductive future [40]. From our study findings, the inclusion of comprehensive education in regular schools, mainly government vernacular schools, is the key recommendation, along with the training of teachers pertaining to SRH by community physicians.

The current study design was ideal for knowing the real effectiveness of an intervention package. Even though the study was conducted during the ongoing COVID-19 pandemic, the study achieved an adequate sample size. Other randomized studies had lesser sample sizes. The intervention package was comprehensive and included all the topics pertaining to SRH; hence intervention was effective in improving KAP among all domains in the intervention arm. Efforts were made by the authors of the study to include all the participants at the follow-up who were present at baseline through phone calls to students and informing parents and teachers regarding the importance of the study, which resulted in less attrition even though the study was conducted during COVID-19 pandemic. Our study demonstrated that schools act as an excellent platform for imparting SRH education.

A few limitations of our study include, the study was conducted only among adolescent girls studying in vernacular (Odia) medium schools. From various study findings, it was observed that adolescent boys had more knowledge of SRH when compared to girls; the former could not be assessed in our study. Our follow-up period was 3 months; hence there is a need for long-term follow-up in further studies. The effectiveness of intervention can be compared between urban and rural areas and in all types of schools, such as private and English medium schools. Our study did not assess the contraceptive practices among adolescent girls; where only the knowledge component was assessed. The gap between the awareness and utilization of contraceptive methods needs to be assessed. Also, the study did not assess sexual activity following intervention which recommends further studies to assess contraceptive usage, sexual activity, and incidence of teenage pregnancies. There are no randomized trials conducted which assessed these domains in an Indian setting owing to cultural taboos. Hence, there is a need for further evidence to strengthen the interventions pertaining to adolescent SRH.

Scale-up assessment: considering the seven key categories for scaling up in the sense of global health, an assessment of the current strategy was done [41]. The National Health Policy of India (NHP), 2017 clearly envisages the provision of sexual health education to adolescents [42]. Thus, the current strategy of implementing comprehensive SRH education in schools has a national focus, scale neutral, with a well-defined scaling strategy for a targeted group of adolescents in any geographic region. The intervention package developed was in accordance with recommendations provided by WHO on comprehensive sexuality education and addresses a significant and persistent problem that is currently high on the agenda of NHP of India. The current strategy resulted in KAP in all domains of SRH with a large effect size when compared to any other study conducted in the Indian state. Each section of the module was prepared after a robust review of the literature, making it both country and state specific to meet the need of adolescent girls. The study was funded by the Indian Council of medical research and the MAMTA Institute for Mother and Child, implicating support for change.

The intervention was delivered through schools, which are already established educational institutions. Training teachers and frontline workers to deliver SRH education could potentially be integrated into existing educational programs. Thus, the model can be implemented with existing educational institutional mechanisms, infrastructure, and human resources, including field functionaries of the health department, NGOs and other private stakeholders engaged in implementing health programs at the micro level, indicating a great fit between intervention and any adopting organization. The intervention provided is a collaborative approach with permission obtained from school mass education. National health programs, namely Ayushmann Bharat [43], envisage outreach visits to schools by healthcare workers to provide counselling services to adolescents. The intervention can be integrated through and scaled out through current programs such as Ayushman Bharat, RKSK, state-owned programs, and various international organizations working on adolescent health with adequate and sustainable funding, a strong network, trained manpower and microlevel institution mechanisms.

## Conclusion

Adolescent girls face various issues pertaining to sexual and reproductive health in day-to-day life. Our study assessed the effectiveness of school-based SRH education among school-going adolescent girls in urban areas of Odisha, India. The study showed a significant increase in knowledge, attitude, and practices pertaining to SRH among students in the intervention arm compared to the control arm. The study has generated evidence that schools can act as a platform for providing SRH education to adolescents, which is an immediate need of the hour in shaping healthy young people for the future. Based on the evidence, policymakers and the Department of School Mass Education should include comprehensive SRH education in the regular school curriculum for adolescents’ health and well-being.

## Data Availability

The datasets used and/or analyzed during the current study are available from the corresponding author upon reasonable request.

## Abbreviations

SRH: Sexual and Reproductive Health
WHO: World Health Organization
UNICEF: United Nations Children’s Fund
CSE: Comprehensive sexuality education
UNFPA: United Nations Population Fund
NFHS: National family health survey
GOI: Government of India
RKSK: Rashtriya Kishore Swasthya Karyakram
AFHC: Adolescent friendly health Clinics
STI: Sexually Transmitted Infections
RTI: Reproductive Tract Infections
HIV/AIDS: Human immunodeficiency virus/acquired immunodeficiency syndrome
KAP: Knowledge, Attitude, and Practices
GOO: Government of Odisha
DEO: District Education Officer
IEC: Institute Ethics Committee
GHS: Government Girls’ High School
MSW: Medico-Social Workers
MS: Microsoft; IBM: International Business Machines
SPSS: Statistical package for social sciences
MHM: Menstrual hygiene management
NHP: National Health Policy
