# Correction: Effectiveness of school-based sexual and reproductive health education among adolescent girls in Urban areas of Odisha, India: a cluster randomized trial

**DOI:** 10.1186/s12978-023-01668-y

**Published:** 2023-09-13

**Authors:** G. Alekhya, Swayam Pragyan Parida, Prajna Paramita Giri, Jasmina Begum, Suravi Patra, Dinesh Prasad Sahu

**Affiliations:** 1 Department of Community Medicine and Family Medicine, AIIMS Bhubaneswar, 3rd Floor, Academic Block, Bhubaneswar, Odisha 751019 India; 2 Department of Obstetrics and Gynaecology, AIIMS Bhubaneswar, Bhubaneswar, Odisha India; 3 Department of Psychiatry, AIIMS Bhubaneswar, Bhubaneswar, Odisha India; 4 WHO NTEP, Bhubaneswar, Odisha India

**Correction: Reproductive Health (2023) 20:105** **https://doi.org/10.1186/s12978-023-01643-7**

After publication of this article [1], it was noted that the legend for Table 3 was inadvertently omitted.

The original article [1] has been corrected.
